# Effect of Prior Local Treatment and Prostate-Specific Antigen Kinetics during Androgen-Deprivation Therapy on the Survival of Castration-Resistant Prostate Cancer

**DOI:** 10.1038/s41598-019-48424-6

**Published:** 2019-08-15

**Authors:** Yoon Soo Hah, Jong Soo Lee, Koon Ho Rha, Sung Joon Hong, Byung Ha Chung, Kyo Chul Koo

**Affiliations:** 1 Department of Urology, Daegu Catholic University Medical Center, Daegu, Republic of Korea; 2 Department of Urology, Severance Hospital, Yonsei University College of Medicine, Seoul, Republic of Korea; 3 Department of Urology, Gangnam Severance Hospital, Yonsei University College of Medicine, Seoul, Republic of Korea

**Keywords:** Prostate cancer, Prostate

## Abstract

Prostate-specific antigen (PSA) kinetics predicts survival in castration-resistant prostate cancer (CRPC); however, the influence of prior treatment on this relationship is unclear. Patients with CRPC were stratified according to time to PSA nadir and time to CRPC progression to investigate their prognostic significance on prostate cancer-specific survival (PCSS) and whether PSA kinetics may serve as prognosticators regardless of prior local treatment. This multicenter retrospective study included 295 patients diagnosed with CRPC between September 2009 and November 2017. PSA kinetics during androgen-deprivation therapy (ADT) including %PSA decline, PSA nadir level, time to PSA nadir, and time to CRPC progression was investigated. Subgroup analysis was performed according to the prior history of local curative treatment. Patients who did not receive prior local treatment with ≥6 months to PSA nadir and <12 months to CRPC, showed lower PCSS rates than those with <6 months to PSA nadir (23.3% vs. 45.3%; *p* = 0.031) and ≥12 months to CRPC (20.0% vs. 47.8%; *p* = 0.001). In patients who had received local treatment, PSA kinetic parameters did not influence PCSS. Our results indicate that time to PSA nadir and time to CRPC progression are prognosticators of PCSS in patients with CRPC who did not previously receive curative local treatment.

## Introduction

Prostate cancer (PCa) shows a protracted natural history with heterogeneous outcomes, depending on the treatment modality^1^. In order to deliver optimal treatment and maximize survival outcomes, it is imperative to develop and validate clinical predictors that can distinguish high-risk patients who may be candidates for active treatment from low-risk patients who can be offered less aggressive therapy.

Prostate-specific antigen (PSA) is the most widely used biomarker of disease burden and treatment response during PCa therapy^2,3^. Observations of the PSA level and of PSA kinetics have been utilized as predictive indicators of disease progression. Controversy exists in relation to the prognostic impact of PSA kinetics on PCa survival. Since the 1990s, several reports have suggested that analysis of PSA kinetics prior to PCa diagnosis could predict tumor grade, stage, and time to disease recurrence following radical prostatectomy (RP)^4–6^. A more diverse approach to the prognostic value of PSA kinetics has been reported since 2000. Studies have analyzed the prognosis of PCa in relation to PSA kinetics prior to treatment, and have suggested that a rapid increase in the PSA level before treatment predicted a relatively high risk of mortality from PCa^7,8^. Moreover, observations of PSA levels after local treatments such as RP or radiation therapy (RT) have reported that PSA doubling time and time to PSA nadir were significantly associated with prostate cancer-specific survival (PCSS)^9–11^. In patients with metastatic PCa treated with androgen deprivation therapy (ADT), a decrease in PSA velocity during ADT was reported to associate with survival^12–16^.

To the best of our knowledge, no study has analyzed the prognostic impact of PSA kinetics during ADT on PCSS in patients stratified according to their prior history of local tumor treatment, and who later received various novel approved agents targeting castration-resistant PCa (CRPC), including abiraterone, enzalutamide, ^233^radium, and cabazitaxel. To address this issue, the present study investigated the utility of PSA kinetics during ADT, and of prior local tumor therapy with curative intent, for the prediction of PCSS in patients diagnosed with CRPC. Despite the approval of various CRPC treatments, and new clinical trials promoting the use of these agents in combination, there is insufficient evidence on which treatment and when to apply such treatment would confer an optimal oncological outcome. Therefore, this specific patient population was selected in order to distinguish high-risk CRPC patients who should be offered active treatment from low-risk patients who may be managed with less aggressive therapy.

## Results

### Baseline clinicopathological characteristics

The clinicopathological features of the patients included in the analysis, stratified by their time to PSA nadir and time to CRPC progression, are described in Table 1. The median age and PSA upon PCa diagnosis were 66.5 years and 55.8 ng/mL, respectively. Among the total of 295 patients, 149 (50.5%) patients underwent prior local treatment: RP in 121 (81.2%) patients and RT in 28 (18.8%) patients. 220 (74.6%) patients had a primary metastatic disease at initial diagnosis, and 75 (25.4%) patients had localized or locally advanced disease. There were no statistically significant differences in the total duration of salvage ADT between patients who received RP and RT (*p* = 0.468).

**Table 1:** Clinicopathological characteristics of patients stratified according to prostate-specific antigen kinetics during androgen-deprivation therapy.

	Overall (n = 295)	Time to PSA nadir			Time to CRPC progression		
		<6 months (n = 218)	≥6 months (n = 77)	*p*	≥12 months (n = 237)	<12 months (n = 58)	*p*
**At initial PCa diagnosis**							
Age (years)	66.5 (61.0–71.8)	66.0 (61.0–71.0)	68.0 (63.0–73.0)	*0.546*	66.5 (61.0–71.0)	66.5 (60.8–72.3)	*0.864*
BMI (kg/m^2^)	24.1 (21.9–25.7)	24.1 (22.1–25.7)	24.1 (21.7–25.7)	*0.823*	24.2 (22.0–25.8)	23.9 (21.8–25.8)	*0.763*
PSA (ng/mL)	55.8 (18.2–335.3)	50.0 (15.0–175.6)	92.9 (26.1–414.4)	*0.445*	53.6 (18.2–212.0)	87.6 (18.0–470.6)	*0.138*
Gleason score				*0.678*			*0.140*
≤7	60 (20.3%)	40 (24.3%)	20 (26.0%)		50 (21.1%)	10 (17.2%)	
≥8	235 (79.7%)	178 (75.7%)	57 (74.0%)		187 (78.9%)	48 (82.8%)	
T stage				*0.194*			*0.009*
≤T2	109 (36.9%)	71 (32.5%)	38 (49.4%)		78 (32.9%)	31 (53.4%)	
≥T3	186 (63.1%)	147 (67.4%)	39 (50.6%)		159 (67.1%)	27 (46.6%)	
N stage				*0.090*			*0.766*
N0	125 (42.4%)	100 (45.9%)	26 (33.8%)		102 (43.0%)	23 (39.7%)	
N1	170 (57.6%)	118 (54.1%)	51 (66.2%)		135 (57.0%)	35 (60.3%)	
M stage				*0.127*			*0.009*
M0	75 (25.4%)	51 (24.4%)	24 (31.2%)		67 (28.3%)	8 (13.8%)	
M1	220 (74.6%)	167 (75.6%)	53 (68.8%)		170 (71.7%)	50 (86.2%)	
**Metastatic site**							
Bone	167 (56.6%)	128 (76.6%)	39 (73.6%)	*0.620*	122 (71.8%)	45 (90.0%)	*0.015*
Visceral	15 (5.1%)	12 (7.2%)	3 (5.7%)	*0.327*	10 (5.9%)	5 (10.0%)	*0.441*
Lymph node	147 (49.8%)	110 (65.9%)	37 (69.8%)	*0.403*	117 (68.8%)	30 (51.7%)	*0.769*
Extent of metastases				*0.708*			*0.915*
<5 sites	120 (54.5%)	94 (56.3%)	26 (49.1%)		91 (53.5%)	29 (58.0%)	
≥5 sites	100 (45.5%)	73 (43.7%)	27 (50.9%)		79 (46.5%)	21 (42.0%)	
Prior local treatment	149 (50.5%)	104 (47.7%)	45 (58.4%)	*0.071*	124 (52.3%)	25 (43.1%)	*0.197*
Radiation therapy	28 (18.8%)	19 (18.3%)	9 (20.0%)	*0.456*	22 (17.7%)	6 (24.0%)	*0.096*
Prostatectomy	121 (81.2%)	85 (81.7%)	36 (80.0%)	*0.486*	102 (82.3%)	19 (76.0%)	*0.246*
**At ADT**							
Initial PSA (ng/mL)	55.9 (18.2–255.2)	23.8 (4.4–125.9)	62.2 (17.2–409.2)	*0.149*	23.6 (3.7–123.6)	68.7 (12.3–327.9)	*0.063*
PSA at nadir (ng/mL)	0.47 (0.10–3.06)	0.54 (0.10–3.14)	0.38 (0.08–3.28)	*0.474*	0.28 (0.07–2.04)	2.55 (0.70–10.3)	*0.468*
Time to nadir (months)	4.0 (2.0–7.0)	3.0 (2.0–4.0)	10.0 (7.0–13.0)	<*0.001*	5.0 (2.0–8.0)	2.0 (1.0–3.0)	<*0.001*
%PSA decline (≥90%)	218 (73.9%)	154 (70.6%)	68 (88.3%)	*0.004*	182 (76.8%)	36 (62.1%)	*0.058*
Time to CRPC (months)	28.0 (13.0–52.0)	11.0 (8.0–17.0)	43.0 (24.5–60.5)	<*0.001*	36.0 (20.0–60.0)	9.0 (6.5–10.0)	<*0.001*
**At CRPC progression**							
PSA (ng/mL)	69.2 (15.0–182.0)	71.8 (15.3–228.3)	58.3 (12.5–154.0)	*0.113*	70.5 (14.9–189.0)	67.6 (17.3–175.7)	*0.805*
Hemoglobin (g/dL)	12.0 (10.7–13.0)	12.2 (10.4–13.1)	11.8 (10.8–12.8)	*0.343*	12.0 (10.6–13.1)	12.1 (10.9–13.0)	*0.371*
Albumin (g/dL)	4.0 (3.7–4.4)	4.0 (3.7–4.4)	4.1 (3.8–4.4)	*0.220*	4.0 (3.7–4.4)	4.1 (3.7–4.5)	*0.530*
ALP (IU/L)	109.0 (70.0–209.0)	119.0 (77.0–241.0)	86.0 (62.5–180.8)	*0.044*	97.5 (68.0–193.0)	156.0 (97.5–406.5)	*0.001*
CCI				*0.013*			*0.240*
≤1	140 (47.5%)	113 (51.8%)	27 (35.1%)		108 (45.6%)	32 (55.2%)	
≥2	155 (52.5%)	105 (48.2%)	50 (64.9%)		129 (54.4%)	26 (44.8%)	
ECOG PS				*0.886*			*0.061*
≤1	159 (53.9%)	127 (58.3%)	32 (41.6%)		124 (52.3%)	35 (60.3%)	
≥2	136 (46.1%)	91 (41.7%)	45 (58.4%)		113 (47.7%)	23 (39.7%)	

Data are median (interquartile range) and number (%).

ADT, androgen deprivation therapy; ALP, alkaline phosphatase; BMI, body mass index; CCI, Charlson Comorbidity Index; CRPC, castration-resistant prostate cancer; ECOG PS, Eastern Cooperative Oncology Group performance status; PCa, prostate cancer; PSA, prostate-specific antigen.

Age, body mass index, PSA, Gleason score, N stage, and metastatic site (except bone) did not differ in the groups stratified by time to PSA nadir or time to CRPC. However, patients with <12 months to CRPC had a lower prevalence of stage ≥T3 disease (*p* = 0.009) and a higher prevalence of metastatic disease at initial PCa diagnosis (*p* = 0.009). At the time of CRPC diagnosis, serum ALP level showed significant differences according to time to PSA nadir (*p* = *0.044*) and time to CRPC (*p* = *0.001*). Patients who had a PSA nadir at ≥6 months exhibited higher CCI than those who had a PSA nadir at <6 months (*p* = 0.013).

Supplementary Table 1 describes the treatments administered following CRPC diagnosis. There were no differences in the proportions of docetaxel, androgen receptor axis-targeted agents, cabazitaxel, and ^233^radium administrations or enrollment in clinical trials between patient subgroups. However, patients with ≥12 months to CRPC received more docetaxel cycles than those with <12 months to CRPC.

### Survival outcomes

**Figure 1:**
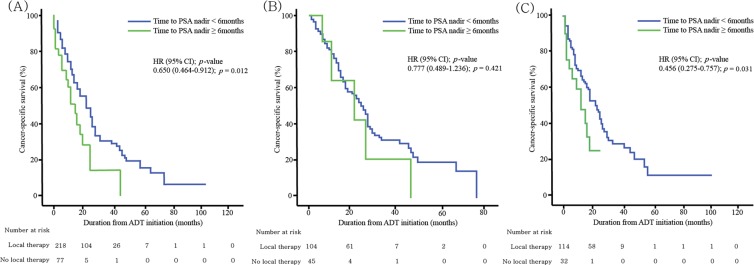
Kaplan-Meier curves showing cumulative cancer-specific survival, stratified by time to PSA nadir (dichotomized at 6 months) in the (**A**) overall group (n = 295), (**B**) patients who had received previous curative local therapy (n = 149), and (**C**) patients who had not received previous curative local therapy (n = 146).

Table 2 shows the outcomes of CRPC patients, stratified according to prior local curative therapy and PSA kinetics during ADT. Figure 1 illustrates PCSS curves, stratified by time to the PSA nadir dichotomized at six months. In patients who had received prior local treatment, there were no difference in the 2-year PCSS outcomes (42.9% vs. 50.4%; *p* = 0.421) (Fig. 1B). However, among patients who did not receive prior curative treatment, men who had PSA nadir at ≥6 months showed lower 2-year PCSS rates than did those with a PSA nadir at <6 months (23.3% vs. 45.3%; *p* = 0.031) (Fig. 1C).

**Table 2:** Survival outcomes of patients stratified according to prior curative local treatment and prostate-specific antigen kinetics.

	Overall (n = 295)	Time to PSA nadir			Time to CRPC progression		
		<6 months (n = 218)	≥6 months (n = 77)	*p*	≥12 months (n = 237)	<12 months (n = 58)	*p*
**PCSS, 2-year (%)**							
Overall group	46.8%	47.0%	28.4%	*0.012*	51.4%	27.4%	<*0.001*
Prior curative local treatment	51.6%	50.4%	42.9%	*0.421*	54.1%	37.6%	*0.017*
No prior curative local treatment	41.2%	45.3%	23.3%	*0.031*	47.8%	20.0%	*0.001*
**CRPC to death period (months)**							
Overall group	16.0 (9.0–27.3)	17.0 (10.0–28.0)	12.5 (5.0–19.5)	*0.118*	17.5 (9.8–28.0)	15.0 (8.0–22.5)	*0.098*
Prior curative local treatment	18.0 (11.0–29.0)	18.0 (11.0–29.0)	19.0 (12.0–31.5)	*0.550*	18.0 (11.0–29.0)	19.0 (13.0–29.0)	*0.851*
No prior curative local treatment	15.0 (7.0–25.0)	15.0 (8.0–26.3)	9.0 (5.0–16.5)	*0.036*	15.0 (8.0–27.3)	12.0 (6.0–18.0)	*0.048*
**Total follow-up period (months)**							
Overall group	19.0 (10.3–29.0)	19.0 (11.0–29.0)	16.0 (9.0–31.5)	*0.738*	20.0 (11.0–32.0)	15.0 (7.5–23.0)	*0.067*
Prior curative local treatment	19.0 (11.3–31.0)	19.0 (12.0–31.0)	13.0 (11.0–28.0)	*0.750*	19.5 (11.0–32.8)	18.0 (13.0–27.8)	*0.260*
No prior curative local treatment	17.0 (8.0–28.0)	19.0 (9.3–29.0)	13.5 (7.5–18.8)	*0.015*	21.0 (10.0–30.0)	12.0 (6.5–20.0)	*0.003*

Data are number (%) and median (IQR).

CRPC, castration-resistant prostate cancer; PCSS, prostate cancer-specific survival; PSA, prostate-specific antigen.

**Figure 2:**
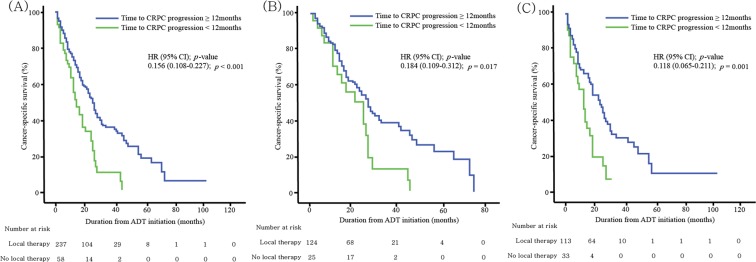
Kaplan-Meier curves showing cumulative cancer-specific survival, stratified by time to castration-resistant prostate cancer (dichotomized at 12 months) in the (**A**) overall group (n = 295), (**B**) patients who had received previous curative local therapy (n = 149), and (**C**) patients who had not received previous curative local therapy (n = 146).

Figure 2 illustrates PCSS curves, stratified by time to CRPC progression dichotomized at 12 months. Lower PCSS rates were observed in patients with CRPC progression at <12 months for both prior local treatment (37.6% vs. 54.1%; *p* = 0.017), and no prior local treatment (20.0% vs. 47.8%; *p* = 0.001) groups.

### Predictors of PCSS

Univariate and multivariate models of predictors of prostate cancer-specific mortality (PCSM) are shown in Table 3. In the overall group, multivariate analysis identified stage ≥T3, Gleason score ≥8, Eastern Cooperative Oncology Group performance status ≥2, serum albumin and ALP levels, time to CRPC <12 months, and no prior local therapy as significant predictors of PCSM. Table 4 shows that in patients who did not undergo curative local therapy, stage ≥T3, Eastern Cooperative Oncology Group performance status ≥2, serum albumin and ALP levels, time to PSA nadir ≥6 months, and time to CRPC <12 months were significant predictors of PCSM. In patients who had received previous curative local therapy, there were no significant relationships between PSA kinetics and PCSM (Table 5).

**Table 3:** Multivariable analyses of prostate cancer-specific mortality in all patients with castration-resistant prostate cancer.

	Univariate			Multivariate		
	HR	(95% CI)	*p*	HR	(95% CI)	*p*
**At PCa diagnosis**						
Age	1.023	(0.996–1.050)	*0.098*			
BMI	0.986	(0.922–1.056)	*0.694*			
T stage (≥T3 vs. ≤T2)	1.563	(1.058–2.309)	*0.025*	1.941	(1.381–2.730)	<*0.001*
N stage (1 vs. 0)	1.629	(0.736–3.645)	*0.226*			
M stage (1 vs. 0)	1.117	(0.739–1.690)	*0.599*			
Metastatic sites (≥5 vs. ≤4)	1.118	(0.791–1.582)	*0.527*			
Gleason score (≥8 vs. ≤7)	1.848	(1.259–2.813)	*0.002*	2.019	(1.433–2.844)	<*0.001*
Prior local therapy (yes vs. no)	0.517	(0.383–0.699)	*<0.001*	0.534	(0.368–0.775)	*0.001*
**PSA kinetics following ADT**						
PSA level at ADT initiation	1.000	(1.000–1.001)	*0.635*			
%PSA decline (≥90%)	0.874	(0.556–1.374)	*0.560*			
PSA nadir level (≥10 ng/ml)	1.328	(0.692–2.549)	*0.394*			
Time to PSA nadir (≥6 months)	1.338	(0.853–2.100)	*0.204*			
Time to CRPC (<12 months)	2.062	(1.458–2.813)	*<0.001*	2.106	(1.157–4.101)	*0.015*
**At CRPC progression**						
PSA	1.000	(1.000–1.001)	*<0.001*	1.000	(1.000–1.001)	*0.425*
Hemoglobin	1.001	(1.000–1.003)	*0.115*			
Albumin	0.468	(0.318–0.688)	*<0.001*	0.674	(0.477–0.952)	*0.025*
ALP	1.001	(1.001–1.002)	*<0.001*	1.001	(1.001–1.001)	<*0.001*
Testosterone	1.278	(0.634–2.412)	*0.345*			
CCI (≥2 vs. ≤1)	1.602	(0.914–2.809)	*0.100*			
ECOG PS (≥2 vs. ≤1)	2.803	(2.232–3.617)	*0.001*	2.968	(1.404–6.277)	*0.004*

ADT, androgen-deprivation therapy; ALP, alkaline phosphatase; BMI, body mass index; CCI, Charlson Comorbidity Index; CI, confidence interval; CRPC, castration-resistant prostate cancer; ECOG PS, Eastern Cooperative Oncology Group performance status; HR, hazards ratio; PCa, prostate cancer; PSA, prostate-specific antigen.

**Table 4:** Multivariable analyses of prostate cancer-specific mortality in patients without a previous history of curative local treatment.

	Univariate			Multivariate		
	HR	(95% CI)	*p*	HR	(95% CI)	*p*
**At PCa diagnosis**						
Age	1.025	(0.995–1.055)	*0.099*			
BMI	0.900	(0.834–0.971)	*0.007*			
T stage (≥T3 vs. ≤T2)	1.783	(1.159–2.741)	*0.008*	1.911	(1.165–3.135)	*0.010*
N stage (1 vs. 0)	1.296	(0.808–2.047)	*0.288*			
M stage (1 vs. 0)	0.574	(0.318–1.035)	*0.065*			
Metastatic sites (≥5 vs. ≤4)	1.009	(0.976–1.042)	*0.607*			
Gleason score (≥8 vs. ≤7)	1.925	(1.272–2.913)	*0.002*	1.489	(0.880–2.522)	*0.138*
**PSA kinetics following ADT**						
PSA level at ADT initiation	1.515	(1.103–2.066)	*0.021*	2.002	(0.993–4.033)	*0.068*
%PSA decline (≥90%)	0.891	(0.555–1.430)	*0.632*			
PSA nadir level (≥10 ng/ml)	1.923	(1.050–3.523)	*0.034*			
Time to PSA nadir (≥6 months)	2.424	(1.771–3.311)	*0.002*	2.501	(1.309–4.778)	*0.006*
Time to CRPC (<12 months)	2.122	(1.326–3.394)	*0.002*	2.768	(1.467–5.223)	*0.002*
**At CRPC progression**						
PSA	1.001	(1.000–1.001)	*0.002*	1.001	(1.000–1.001)	*0.070*
Hemoglobin	0.782	(0.704–0.868)	*0.001*	0.906	(0.771–1.063)	*0.225*
Albumin	0.335	(0.230–0.488)	<*0.001*	0.333	(0.199–0.558)	*0.001*
ALP	1.002	(1.001–1.003)	<*0.001*	1.002	(1.001–1.003)	*0.002*
Testosterone	1.338	(0.749–2.716)	*0.147*			
CCI (≥2 vs. ≤1)	1.266	(0.846–1.895)	*0.251*			
ECOG PS (≥2 vs. ≤1)	2.854	(1.329–4.813)	<*0.001*	2.193	(1.023–4.700)	*0.044*

ADT, androgen-deprivation therapy; ALP, alkaline phosphatase; BMI, body mass index; CCI, Charlson Comorbidity Index; CI, confidence interval; CRPC, castration-resistant prostate cancer; ECOG PS, Eastern Cooperative Oncology Group performance status; HR, hazards ratio; PCa, prostate cancer; PSA, prostate-specific antigen.

**Table 5:** Multivariable analyses of prostate cancer-specific mortality in patients with a previous history of curative local treatment.

	Univariate			Multivariate		
	HR	(95% CI)	*p*	HR	(95% CI)	*p*
**At PCa diagnosis**						
Age	1.020	(0.979–1.063)	*0.334*			
BMI	1.092	(0.981–1.216)	*0.108*			
T stage (≥T3 vs. ≤T2)	1.336	(0.728–2.455)	*0.350*			
N stage (1 vs. 0)	0.559	(0.162–1.890)	*0.367*			
M stage (1 vs. 0)	1.370	(0.866–2.165)	*0.178*			
Metastatic sites (≥5 vs. ≤4)	1.035	(0.585–1.831)	*0.906*			
Gleason score (≥8 vs. ≤7)	2.253	(1.156–4.390)	*0.017*	2.949	(1.412–6.160)	*0.004*
**PSA kinetics following ADT**						
PSA level at ADT initiation	0.999	(0.998–1.001)	*0.216*			
%PSA decline (≥90%)	0.657	(0.338–1.275)	*0.657*			
PSA nadir level (≥10 ng/ml)	1.441	(0.582–3.656)	*0.429*			
Time to PSA nadir (≥6 months)	1.969	(0.924–4.192)	*0.079*			
Time to CRPC (<12 months)	1.832	(1.100–3.050)	*0.020*	1.225	(0.687–2.185)	*0.492*
**At CRPC progression**						
PSA	1.001	(1.000–1.001)	*<0.001*	1.003	(1.001–1.005)	*0.002*
Hemoglobin	1.002	(1.000–1.003)	*0.035*			
Albumin	0.641	(0.326–1.261)	*0.198*			
ALP	1.002	(1.001–1.003)	*0.002*	2.090	(0.940–4.647)	*0.071*
Testosterone	1.435	(0.567–1.944)	*0.474*			
CCI (≥2 vs. ≤1)	0.829	(0.445–1.584)	*0.839*			
ECOG PS (≥2 vs. ≤1)	2.446	(1.051–5.692)	*0.038*	3.966	(1.270–12.38)	*0.018*

ADT, androgen-deprivation therapy; ALP, alkaline phosphatase; BMI, body mass index; CCI, Charlson Comorbidity Index; CI, confidence interval; CRPC, castration-resistant prostate cancer; ECOG PS, Eastern Cooperative Oncology Group performance status; HR, hazards ratio; PCa, prostate cancer; PSA, prostate-specific antigen.

## Discussion

A comprehensive set of clinicopathological factors at ADT initiation and PSA kinetics observed during ADT, were analyzed to investigate predictors of PCSS in patients who later developed CRPC. In the current era of multimodal therapies targeted at CRPC, we focused on this specific population in order to deliver an optimal treatment strategy for individual patients. Since disease progression may be affected by the treatment of the primary tumor, patients were stratified according to their previous history of curative local treatment. In patients who had not received curative local treatment, those with PSA nadir at ≥6 months and time to CRPC of <12 months showed lower rates of 2-year PCSS, compared to patients with PSA nadir at <6 months and time to CRPC of ≥12 months. However, in patients who had received curative local treatment, time to PSA nadir did not influence PCSS. Patients who are at high risk of disease progression should be identified at an early stage using PSA kinetics and history of curative local treatment and receive active treatment, while low-risk patients can be managed with less aggressive therapy.

Multimodal strategies exist for PCa treatment, ranging from active surveillance to aggressive combined therapies, with heterogeneous survival outcomes. PSA kinetics is considered to represent a prognostic marker of PCa progression. Many previous reports have investigated PSA kinetics in patients receiving systemic therapies for BCR and metastatic PCa. However, to our knowledge, our study is the first to consider whether a prior history of curative local treatment influences the prognostic value of PSA kinetics, after adjusting for a comprehensive set of clinicopathological data. A noteworthy finding was the PCSS benefit observed with local treatment in patients with CRPC. Our results add to existing literature that local treatments targeted at the primary tumor in patients with castration-naïve PCa may confer a survival benefit in the disease course of CRPC progression. Still, a cautious interpretation would be warranted in that 25.4% of our cohort did not exhibit *de novo* metastasis at the diagnosis of PCa.

Some of our findings contrasted with those of previous studies. Several previous reports suggested that PSA kinetic parameters such as PSA doubling time and time to PSA nadir were significant predictors of survival in patients who had received curative treatment. However, these studies were limited by the differences in patient baseline characteristics and the absence of a clear definition of BCR, which is an important criterion for ADT initiation following local therapy. Zhou *et al*. proposed PSA doubling time and Gleason score as predictors of PCSM following RP or RT^9^. Of note, most of the patients were in the early stages of the disease with favorable Gleason score and T stage compared to our cohort. Furthermore, the PSA level used to confirm ‘PSA-defined recurrence’ was not clearly specified. The median PSA level upon ADT initiation was 9.4 ng/mL in RP patients and 9.8 ng/mL in RT patients. D’Amico *et al*. also identified an association between time to PSA nadir and PCSM in patients with BCR after local therapy^11^. In this study, more than 50% of the patients exhibited Gleason score ≤6. In contrast, our patients exhibited higher Gleason score and TNM stage since the retrospective data collection was limited to patients who later developed CRPC. The PSA working group criteria for PSA failure were used in the D’Amico *et al*. study, rather than the 2018 EAU-ESTRO-ESUR-SIOG guidelines^17,18^. Moreover, patients with >20 ng/mL PSA at the initiation of ADT were also included. Despite the controversy regarding the optimal PSA value to define BCR, it would be arduous to apply these findings to contemporary patients^19,20^. This implies that the PSA cutoff used to define BCR sensitively influences prognostic data interpretation. In the present study, all patients were followed and managed according to contemporary treatment guidelines, suggesting that our results are generalizable to the contemporary PCa population.

The relationship between time to PSA nadir and survival outcome is controversial. Some studies reported that shorter time to PSA nadir associated with improved PCSS^11,15,21^. In contrast, others reported that shorter time to PSA nadir was associated with reduced overall survival^14,16,22,23^. A rapid decline of PSA during ADT is generally considered to correlate with more hormone sensitivity in the PCa cells, and less development of androgen-independent PCa cells, accompanied by a longer remission period and improved survival^21^. One of the reasons for the contrary results is presumed to be the ablation of the androgen receptor, which acts as a tumor suppressor^13^. However, the relationship between this molecular tumor feature and overall survival has not been fully elucidated. Moreover, the endpoint of this cited study was sequential increases in PSA levels, rather than survival. Thus, direct comparisons cannot be made between these studies due to differences in patient characteristics, BCR definitions, and primary endpoints.

Our study was strengthened by the incorporation of a wide range of potential prognosticators, the availability of detailed clinicopathologic data, and information on treatment outcomes, which were available from all patients. Most importantly, the independent survival prognosticators identified here are those readily available in everyday clinical practice; this increases their general applicability. At the same time, we acknowledge several limitations of the study. First, this was a retrospective study and sampling intervals used to estimate PSA kinetics during ADT were not therefore defined prospectively or standardized. Second, the treatments administered were not uniformly selected. Although there were no statistically significant differences between the treatments employed for CRPC, the possibility of bias due to subgroup differences cannot be overlooked. For instance, there was a difference in the number of docetaxel cycles administered to patients stratified according to the time to CRPC. This suggests that there may have been greater chemo-tolerance in patients who received more chemotherapeutic cycles, which might have affected our results. Third, as our study population included patients who were relatively older and had multiple comorbidities, PCSS was chosen as the end point of our study instead of overall survival. Lastly, the treatment paradigm for castration sensitive PCa is rapidly evolving with accumulating evidence that upfront chemotherapy and androgen receptor axis-targeted agents may improve PCSS. Therefore, the results of our study may not apply to patients receiving these multimodal treatments. Despite these limitations, our study provides meaningful data indicating that PSA kinetics cannot be utilized as an independent prognostic marker in all PCa patients and should be interpreted in the context of previous curative local therapy.

In conclusion, time to PSA nadir and time to CRPC onset during ADT are prognosticators of PCSS in patients with CRPC who did not previously receive local curative treatment. In contrast, PSA kinetics may not be useful as a prognosticator of PCSS in patients who have received curative local therapy. Further larger scale analyses are warranted to define the influence of local treatment on ADT pharmacokinetics and on the future response to CRPC treatment.

## Methods

### Patient selection and data collection

Clinicopathological data were collected from two Korean institutions: Severance Hospital and Gangnam Severance Hospital (Yonsei University Health System). A total of 295 consecutive patients who were diagnosed with CRPC progression between September 2009 and November 2017 were included in this multicenter retrospective study.

**Figure 3:**
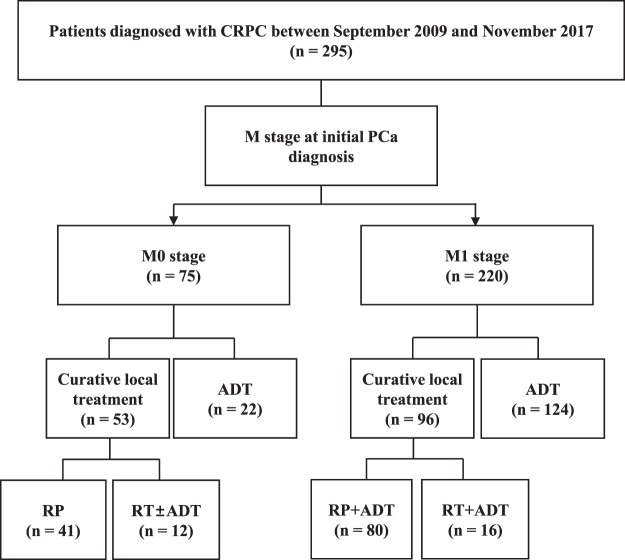
Flow diagram of the patient cohort involved in this study. ADT, androgen deprivation therapy; CRPC, castration-resistance prostate cancer; PCa, prostate cancer; RP, radical prostatectomy; RT, radiation therapy

Patients with *de novo* metastasis were given ADT as initial treatment, and no patient was offered intermittent ADT. RP or RTx with or without ADT were performed for patients with localized or locally advanced disease, and those with a limited metastatic burden. Surgery was performed by the retropubic or robotic approach, with the extent of pelvic lymph node dissection based upon the risk category of the patient. RT consisted of intensity modulated external beam RT with the median RT dose of 7,000 cGy (IQR 7,000 to 7,000) (Fig. 3).

The criteria for confirming biochemical recurrence (BCR) after receiving RP included a PSA level of ≥0.2 ng/mL^24,25^. Patients exhibiting BCR following RP without radiologic recurrence received salvage RT with short-term ADT. Long-term ADT was initiated following further radiographic disease progression. BCR following RT was defined as a rise of ≥2.0 ng/mL above the nadir^26^. ADT was initiated following BCR. The CRPC diagnostic criteria followed the recommendation of the 2018 EAU-ESTRO-ESUR-SIOG Guidelines on Prostate Cancer^18^. These criteria were: castrate serum testosterone <50 ng/dL or 1.7 nmol/L plus either; (a) biochemical progression: three consecutive rises in PSA one week apart resulting in two 50% increases over the nadir, and a PSA >2.0 ng/mL, or (b) radiological progression: the appearance of new lesions: either two or more new bone lesions on bone scan or a soft tissue lesion using Response Evaluation Criteria in Solid Tumours^27^. This retrospective study was approved by Institutional Review Board of Yonsei University (2017-0186-001), which waived the requirement for informed consent. All study procedures complied with the principles of the 1946 Declaration of Helsinki, and its 2008 update.

Clinicopathological data included age, body mass index, PSA, Gleason score, T stage, N stage, M stage, and metastatic sites at the time of initial PCa diagnosis. PSA kinetics analyzed included the PSA nadir level, time to PSA nadir, %PSA decline, and time to CRPC, based on the initial PSA at the time of ADT initiation. Serum PSA, hemoglobin, albumin, and alkaline phosphatase (ALP) levels, the Charlson comorbidity index, and Eastern Cooperative Oncology Group performance status score were collected at CRPC diagnosis.

Patients with CRPC were stratified according to time to PSA nadir dichotomized at six months, and time to CRPC progression dichotomized at 12 months to investigate the prognostic significance of PSA kinetics on PCSS. Subgroup analyses were performed according to the prior history of local curative treatment.

### Statistical analyses

The study groups were compared using the two-sided Mann–Whitney U test (for the analysis of continuous variables) and the chi-square test (for the analysis of two or more variables). Variables considered as potential predictors for multivariate modeling were selected by univariate analyses using the Cox-proportional hazards regression model. The optimal cut-off value for each PSA kinetic parameter was determined by reference to the maximum of the Youden index in each receiver operating characteristic curve^28^.

To compare the survival outcomes of each patient group, Kaplan-Meier survival analysis was performed according to time to PSA nadir and time to CRPC, dichotomized at 6 and 12 months, respectively. All statistical analyses were performed using IBM SPSS software (version 21.0; IBM Corporation, Armonk, NY, USA) and R statistical package (version 3.2.0; Institute for statistics and mathematics, Vienna, Austria). Differences with a *p*-value of <0.05 were considered statistically significant.

## Supplementary information

Supplementary table
