# Supplementary material for: Effect of Prior Local Treatment and Prostate-Specific Antigen Kinetics during Androgen-Deprivation Therapy on the Survival of Castration-Resistant Prostate Cancer
Source: Sci Rep. 2019 Aug 15;9:11899. doi: 10.1038/s41598-019-48424-6 (PMC6695395; doi:10.1038/s41598-019-48424-6)
Supplement: Supplementary file 1 — Supplementary table [file 41598_2019_48424_MOESM1_ESM.docx]

**Supporting Information**

**Effect of Prior Local Treatment and Prostate-Specific Antigen Kinetics during Androgen-Deprivation Therapy on the Survival of Castration-Resistant Prostate Cancer**

^1^Yoon Soo Hah, ^2^Jong Soo Lee, ^2^Koon Ho Rha, ^2^Sung Joon Hong, ^3^Byung Ha Chung, ^3*^Kyo Chul Koo

^1^Department of Urology, Daegu Catholic University Medical Center, Daegu, Republic of Korea

^2^Department of Urology, Severance Hospital, Yonsei University College of Medicine, Seoul, Republic of Korea

^3^Department of Urology, Gangnam Severance Hospital, Yonsei University College of Medicine, Seoul, Republic of Korea

**^*^Corresponding author:** Kyo Chul Koo, MD, PhD

Department of Urology, Gangnam Severance Hospital, Yonsei University College of Medicine, 211 Eonju-ro, Gangnam-gu, Seoul, Republic of Korea, 135-720.

Tel: +82-2-2019-3470

Fax: +82-2-3462-8887

E-mail: gckoo@yuhs.ac

Supplementary table. Treatments administered for castration-resistant prostate cancer.

|  | **Overall**  **(n = 295)** | **Time to PSA nadir** | | |  | **Time to CRPC progression** | | |
| --- | --- | --- | --- | --- | --- | --- | --- | --- |
|  |  | **<6 months**  **(n = 218)** | **≥6 months**  **(n = 77)** | ***p*** |  | **≥12 months**  **(n = 237)** | **<12 months**  **(n = 58)** | ***p*** |
| Docetaxel |  |  |  |  |  |  |  |  |
| N | 264 (89.5%) | 196 (89.9%) | 68 (88.3%) | 0.670 |  | 216 (91.1%) | 48 (82.8%) | 0.091 |
| No. cycles | 4.0 (3.0 – 7.5) | 5.0 (3.0 – 9.5) | 3.0 (2.5 – 5.0) | 0.054 |  | 5.0 (3.0 – 8.5) | 3.0 (2.5 – 5.5) | 0.028 |
| ARAT agent use | 201 (68.1%) | 155 (71.1%) | 46 (59.7%) | 0.087 |  | 158 (66.7%) | 43 (74.1%) | 0.346 |
| Pre-chemotherapy | 33 (11.2%) | 20 (9.2%) | 13 (16.9%) | 0.090 |  | 28 (11.8%) | 5 (8.6%) | 0.643 |
| Abiraterone | 14 (4.7%) | 8 (3.7%) | 6 (7.8%) | 0.208 |  | 9 (3.8%) | 5 (8.6%) | 0.161 |
| Enzalutamide | 27 (9.2%) | 17 (7.8%) | 10 (13.0%) | 0.175 |  | 23 (9.7%) | 4 (6.9%) | 0.619 |
| Post-chemotherapy | 154 (52.2%) | 120 (55.0%) | 34 (44.2%) | 0.112 |  | 126 (53.2%) | 28 (48.3%) | 0.559 |
| Abiraterone | 38 (12.9%) | 24 (11.0%) | 14 (18.2%) | 0.116 |  | 33 (13.9%) | 5 (8.6%) | 0.382 |
| Enzalutamide | 134 (45.4%) | 102 (46.8%) | 32 (41.6%) | 0.506 |  | 111 (46.8%) | 23 (39.7%) | 0.378 |
| Cabazitaxel | 4 (1.4%) | 2 (0.9%) | 2 (2.6%) | 0.280 |  | 3 (1.3%) | 1 (1.7%) | 0.586 |
| ^233^Radium | 2 (0.7%) | 1 (0.5%) | 1 (1.3%) | 0.455 |  | 2 (0.8%) | 0 (0.0%) | 1.000 |
| Clinical trials | 73 (24.7%) | 57 (26.1%) | 16 (20.8%) | 0.443 |  | 59 (24.9%) | 14 (24.1%) | 1.000 |

Data are median (interquartile range) and number (%)

ARAT, androgen receptor-axis targeted; PSA, prostate-specific antigen
